# Bone Mineral Density in Gravida: Effect of Pregnancies and Breast-Feeding in Women of Differing Ages and Parity

**DOI:** 10.1155/2014/897182

**Published:** 2014-11-20

**Authors:** Ehud Lebel, Yuri Mishukov, Liana Babchenko, Arnon Samueloff, Ari Zimran, Deborah Elstein

**Affiliations:** ^1^Department of Orthopedic Surgery, Shaare Zedek Medical Center Affiliated with the Hebrew University Hadassah School of Medicine, P.O. Box 3235, 12 Bayit Street, 91031 Jerusalem, Israel; ^2^Gaucher Clinic, Shaare Zedek Medical Center Affiliated with the Hebrew University Hadassah School of Medicine, P.O. Box 3235, 12 Bayit Street, 91031 Jerusalem, Israel; ^3^Department of Obstetrics & Gynecology, Shaare Zedek Medical Center Affiliated with the Hebrew University Hadassah School of Medicine, P.O. Box 3235, 12 Bayit Street, 91031 Jerusalem, Israel

## Abstract

Changes of bone during pregnancy and during lactation evaluated by bone mineral density (BMD) may have implications for risk of osteoporosis and fractures. We studied BMD in women of differing ages, parity, and lactation histories immediately postpartum for BMD, *T*-scores, and *Z*-scores. Institutional Review Board approval was received. All women while still in hospital postpartum were asked to participate. BMD was performed by dual-energy X-ray absorptiometry (DXA) machine at femoral neck (FN) and lumbar spine (LS) by a single technician. Of 132 participants, 73 (55.3%) were ≤30 years; 27 (20.5%) were primiparous; 36 (27.3%) were grand multiparous; 35 (26.5%) never breast fed. Mean FN *T*-scores and *Z*-scores were higher than respective mean LS scores, but all means were within the normal limits. Mean LS *T*-scores and *Z*-scores were highest in the grand multiparas. There were only 2 (1.5%) outliers with low *Z*-scores. We conclude that, in a large cohort of Israeli women with BMD parameters assessed by DXA within two days postpartum, mean *T*-scores and *Z*-scores at both the LS and FN were within normal limits regardless of age (20–46 years), parity (1–13 viable births), and history of either no or prolonged months of lactation (up to 11.25 years).

## 1. Introduction

Concerns exist regarding changes in the structure and metabolism of bone during pregnancy and the early postpartum period including during lactation that may have implications for short-term and long-term risk of osteoporosis and fractures. Since calcium mobilization [1] and bone resorption increase at the end of pregnancy and increase further with lactation [2], there has been considerable controversy over the past decades as to whether high parity and/or prolonged lactation periods are detrimental to bone mineral density (BMD). Although there is loss of BMD during pregnancy [3] and during lactation [4], it may be considered to be transient. Yet it has also been suggested that factors such as age at and duration of menopause [5–7] may be the more definitive risk factors for osteoporosis and fractures.

Recently, it has been shown that pregnancies [8] and long or repeated periods of breast-feeding do not seem to be associated with emergence of osteoporosis in later life [7, 9]. In fact, women with multiple pregnancies have been shown to have the same or higher BMD [10] and lower fracture risk decades after last parturition [6, 7] when compared with nulliparous women.

International studies in various populations of women with high parity [10, 11] and motivated to repeated periods of lactation generally confirm good recovery of BMD but these studies are skewed because of comparisons to nulliparous but younger control groups. In a study of >200 Sri Lankan women, women with ≥5 children and women who had breast fed for ≥97 months had an age-adjusted BMD at lumbar spine (LS) and femoral neck (FN) that were not significantly different from that of women with lesser parity and fewer months of lactation [12].

The purpose of the current study is to evaluate BMD in Israeli women of differing parity and lactation histories immediately postpartum. The following question will be addressed: is there a quantitative and/or qualitative difference in BMD (based on *T*-scores and *Z*-scores) at FN and/or LS (in women who are not yet menopausal) based on the cumulative months of pregnancies and approximate cumulative months of breast-feeding based on parity and/or age?

## 2. Method

All women still in hospital postpartum (up to 48 hours) were asked to participate in this study and were offered a free evaluation of BMD and, if desired, consultation with an osteoporosis expert. In this entailed appearing for the examination at a specific time and on a different floor from the obstetrics department, some women were unable to participate (e.g., the logistics of having the baby lying-in).

All candidates were asked to complete a questionnaire regarding demographic characteristics including weight before pregnancy and postpartum, height, diet, allergies, previous abortions, smoking history, and comorbidities including gestational diabetes, approximate cumulative months of pregnancies, and approximate cumulative months of breast-feeding after previous births.

*Post hoc,* for statistical analyses, the cohort was subdivided according to parity into four groups: primiparous (no successful pregnancies previous to the current one), low-parity (2nd–5th live births); medium parity or grand multiparous (6th–9th live births), and grand-grand multiparous (GGMP: ≥10th live birth).

BMD evaluation was performed on a standard dual-energy X-ray absorptiometry machine (DXA; Hologic, Bedford, MA, USA) by a single technician while the mother was still in hospital. Results were recorded as BMD and as *T*-scores (compared to healthy females 25–30 years old and who are categorized according to WHO classifications to identify persons who are at risk for osteoporosis and/or fractures) and *Z*-scores (compared to healthy age-matched females) at the FN and LS.

### 2.1. Statistical Analysis

Comparisons of means of parity groups used Levene's test and *t*-test for independent variables (age, height, weight, BMI, use of medications and dietary supplements, and duration of lactation). Analysis of variance was used to evaluate homogeneity of these variables within and among parity groups. Results of bone density ((BMD) *T*-score and *Z*-score of each skeletal domain) were compared and correlated with each of the independent variables, using Pearson's correlation coefficient. The same procedure was used for evaluation of the relationship between bone density and duration of lactation. Two-tailed significance level was set at *P* ≤ 0.05. All analyses were performed by IBM SPSS 20.0.

## 3. Results

Institutional Review Board (Helsinki Committee) approval for this study was received and all participants signed Informed Consent for a questionnaire and for performance of a DXA examination (gratis).

There were 132 women who underwent DXA evaluation, 128 (97.0%) of whom had singleton births.

Table 1 presents the demographic data based on answers provided in the questionnaire and BMD outcomes of the whole group. There were fewer than 7% of women with a history of smoking (at any time) and fewer than 4% of women whose mother had been diagnosed with osteoporosis. There were very few outliers for prepregnancy body weight and body mass index (BMI). In general, mean FN *T*-scores and mean *Z*-scores were higher than mean *T*-scores and mean *Z*-scores at the LS, but all means were within the normal range.

Table 2 presents the descriptive data of the group when it was subdivided into women who were ≤30 years (*n* = 73) and women >30 years of age (*n* = 59). There were significant differences between groups for gravidity, parity, months of pregnancies (where 8.5 was used as an approximate mean for pregnancy months), and months of lactation (*P* = 0.000). As in the whole group, mean *T*-scores and mean *Z*-scores were higher at the FN than LS, respectively, in the younger women with mean *Z*-scores lower than mean *T*-scores. In this case, *T*-scores in the younger women may be misleading because, by definition, many of these women had not yet achieved peak bone mass (i.e., they were younger than 25–30 years of age which is the standard for *T*-scores) while theoretically all of the older women had achieved peak bone mass. This may be the reason why the mean *T*-scores at each site were not significantly different from the mean *Z*-scores in the older women only.

Table 3 presents the group subdivided by parity, that is, comparing primiparous (and hence also no lactation history) relative to all the multiparous women (with a spectrum of parity and months of lactation including no lactation experience in 8 women). There were no statistically significant differences between the primiparous group and the multiparous group with regard to any of the bone density measures.

Table 4 presents the descriptive statistics of the cohort when subdivided into subgroups based on the predefined parity groups: Group #1 is primiparous; Group #2 is 2–5 children; Group #3 (grand multiparous) is ≥6 children of which 6 women had ≥10 viable births (grand-grand multiparous; GGMP). With regard to mean *T*-scores at the FN versus mean *T*-scores at the LS, there were significant but opposite trends: mean FN *T*-scores were the highest in the primiparous and the lowest in the grand multiparous whereas mean LS *T*-scores and mean *Z*-scores were highest in the primiparous and lowest in the grand multiparous. Mean FN *Z*-scores were the same in all groups.

The maternal age is a background factor that might be closely related to other independent factors such as parity and cumulative time of breast-feeding.* Post hoc,* this assumption was evaluated by the Pearson correlation test and was indeed found to have a relatively high correlation coefficient (*r* = 0.583). Therefore, in an attempt to tease out the effect of each of these factors on bone density separately, we evaluated the effects of these factors (*β*), as well as the prediction power (*R*) in a linear regression, where we also tested for colinearity by the variance inflation factor (VIF) test. These tests did not reveal substantial colinearity (VIF < 5). Thus, we assume that these factors have unique effects.

Table 5 presents the results of the BMD data for women who never breast fed (*n* = 35) relative to those who had varying histories of lactation (up to 135 months). There was a significantly higher mean FN *T*-score and *Z*-score among those who never breast fed but no difference in mean LS *T*-scores or mean *Z*-scores which, as in the whole group, were lower than the respective FN scores.

There were 12 outliers (9.1%) based on *T*-scores and/or *Z*-scores of <−2.0 at the LS; none of the women had *T*-scores or *Z*-scores at the FN <−2.0. Only one of these (8.3%) reported a fracture during childhood. Their characteristics are presented in Table 6. Of these 12 women, only three (2.3% of the entire cohort) had *T*-scores or *Z*-scores <−2.5. They were aged 29, 30, and 33 years; prepregnancy weight was 41, 55, and 90 kgs, respectively; they had 5, 5, and 8 children, respectively, with 44, 24, and 98 months of lactation, respectively. The last woman (aged 33 years, prepregnancy weight 90 kg, with 8 children) had borderline values of [−2.5]*T*-score and [−2.4]*Z*-score. Thus, the two other women, who would be considered at risk of fractures/osteoporosis (and indeed the thinner woman was the single outlier with a fracture in the past), were in the median of the group for age and weight and had 5 children each and neither the least or the greatest months of breast-feeding/pregnancies.

## 4. Discussion

To date, there is only one study that includes both cross-sectional and longitudinal data on postpartum women and BMD measures. That study investigated BMD in women who had just given birth and showed that there is a loss of bone density at the LS of 7.6% relative to age-matched, nonpostpartum controls but also showed the decreased bone density to be transient [13]. In addition, a longitudinal evaluation in premenopausal women [13] comparing those with parity of 4–7 births relative women with 0–2 births showed that breast-feeding for 1–6 months decreased FN BMD by 2% postpartum but that no further BMD loss occurred after 5 months postpartum. Although absolute BMD values are not indicative of risk of fractures/osteoporosis like *T*-scores and, in addition, BMD is not reflective of confounding variables among age-matched individuals like *Z*-scores, these authors concluded that the BMD loss was transient and neither an extended lactation period (which in this study was up to 6 months only) nor multiple pregnancies (which was up to 7 births only) were a risk factor when predicting women at risk for future osteoporosis. These conclusions also are comparable to those of other studies in older women with grand multiparity and extended periods of lactation where BMD at multiple sites was not worse than in nulliparous older women [11, 14, 15].

The most important new finding derived from the current study, which (a) included women with greater parity and (b) women with very extended lactation histories compared to those that have heretofore been studied and also (c) compared primiparous relative to multiparous women, was that the mean values of all the bone density measures taken directly after birth were well within the normal ranges. The mean *T*-scores and *Z*-scores were within normal values regardless of maternal age being older (>30 years of age) relative to younger (≤30 years of age), regardless of whether the mother was primiparous or multiparous including women who had had up to 13 births; and, similarly, the number of months/years of lactation did not result in diminution of mean BMD *T*-scores into the osteopenic category. Even when evaluating the outliers for *T*-scores and/or *Z*-scores in the [−2.5] range, only two women were identified and, in these, both *T*-scores and *Z*-scores were <[−2.5] but the clinical characteristics of the women were not indicative of their low bone density status.

A second important finding was that *T*-scores and *Z*-scores at the FN were invariably higher than the respective LS scores, again regardless of the maternal age, parity, and/or lactation history. A partial explanation may be that classically the FN has not been used as a preferred site for assessment of fracture risk and/or osteoporosis because biological processes affecting bone extend from the proximal to the more distal sites, and hence earlier detection of pathology would be at the lumbar spine. For example, in the full cohort of women, FN *T*-scores and *Z*-scores were −0.49 and −0.39, respectively, but the values at the LS were −0.74 and −0.92, respectively. Comparable differences can be seen when comparing the subgroups by age, parity, and lactation history; none of these scores, however, resulted in mean *T*-scores or *Z*-scores >1.0 standard deviation below normal.

Nonetheless, although most studies in older women use LS *T*-scores and *Z*-scores to assess fracture risk and bone health, in pregnant women, one might also be concerned about transient osteoporosis of the hip (TOH) of pregnancy. During the course of this survey, there was no case of TOH which most often develops in the third trimester and resolves by six months postpartum [16]. In general hip pathology during pregnancy is rare, as confirmed in a 2-year prospective survey of nearly 5000 pregnancies in France, where there was only one case of TOH and in a parallel 15-year retrospective study where only 6 patients (9 hips) with TOH were identified [17]; of the latter six women, five had osteopenia based on DXA at the LS. Thus, despite inclusion in the current study of some women with advanced maternal age and/or high parity and/or extended lactation, TOH was not seen in the current cohort. There were also no cases of fractures in these women during the current pregnancy: all fractures reported by the women were noted prior to child-bearing years. However, one might consider that the two women (1.5%) who were identified as having *T*-scores and *Z*-scores at the LS in the osteoporotic range (but were otherwise not outliers for any of the clinical characteristics) might actually have mild TOH based on guidelines in the literature, but this is unlikely, and, moreover, the FN scores in these two women were not outliers.

Importantly, because of the ability to further subdivide the multiparous cohort into two groups, some subtle but potentially clinically meaningful differences were uncovered despite the lack of statistical significance. Specifically, the mean LS *T*-scores and mean *Z*-scores were highest in the grand multiparas, slightly lower in the multiparas, and the lowest in the primiparas Because most previous studies only compared primiparas to multiparas and rarely had included many grand-grand multiparous women, this new information may be valuable in confirming the hypothesis that increased parity does not negatively impact bone density.

Similarly, with regard to lactation history, no previous studies included women with very extended lactation histories (albeit who naturally also had multiple pregnancies). Interestingly, in comparison to women who never breast fed, there was no statistically significant difference in LS measures (which heretofore has been the basis of assessment of fracture risk and osteoporosis in comparative studies of nulliparous to multiparous postmenopausal women) while there were significant differences in the FN bone measures.

The limitation of this study, if indeed it is a limitation, is that Israeli women are encouraged to achieve their personal maximal family size and are also supported by well-baby clinics in maintaining good health standards prenatally. This is indirectly seen in the numbers of women complying with prenatal vitamins, not smoking, and so on. Thus, from the point of view of nutrition and prenatal care, most Israeli women, regardless of ethnicity, are healthy. On the other hand, very few claimed to have mothers with osteoporosis which may indicate that osteoporosis, which is a multifactorial trait, may be less common in this cohort; a more likely explanation is that most of their mothers are too young to have evidence of osteoporosis.

Of relevance as well are recent data regarding the long-term effect of parity on BMD which confirm that parity greater than 7 births seems to have an osteoprotective effect against age-related bone loss in postmenopausal women [8].

In conclusion, in a large cohort of Israeli women whose bone density parameters were assessed by DXA within the first two days postpartum, mean *T*-scores and *Z*-scores at both the lumbar spine and femoral neck were within the normal range regardless of age (20–46 years), parity (1–13 viable births), and history of lactation (up to 11.25 years). In total, only two women (1.5%) were identified as having both *T*-scores and *Z*-scores at the LS (but not FN) in the osteoporotic range, yet they were otherwise not outliers for any of the clinical characteristics.

## Figures and Tables

**Table 1:** Demographic data of the cohort (*n* = 132).

Maternal age mean ± SD (range)	29.86 ± 5.97 (20–46) years
Height mean ± SD (range)	162.49 ± 6.34 (145–179) cms
Prepregnancy weight mean ± SD (range)	63.98 ± 12.97 (41–12) kg
Prepregnancy BMI mean ± SD (range)	24.17 ± 4.36 (16.8–41.5)
Current weight mean ± SD (range)	71.86 ± 14.04 (43–125) kg
Current BMI mean ± SD (range)	27.15 ± 4.62 (18.0–40.5)
Weight gain mean ± SD (range)	8.37 ± 7.42 ([−9]–67) kg
Gravidity mean ± SD (range)	4.05 ± 2.74 (1–13)
Parity mean ± SD (range)	3.92 ± 2.62 (1–13)
Months lactation mean ± SD (range)	27.09 ± 31.61 (0–135.0) months
Months pregnancy + lactation mean ± SD (range)	60.13 ± 51.79 (8.5–220.0) months
Pregnancies using *in vitro* fertilization (IVF)	8 (6.1%)
Twin births	4 (3.0%)
Previous missed abortion	15 (11.4%)
Singletons' birth weight (range)	3306 (2460–5500) gms
Use of prenatal vitamins	64 (48.5%)
Use of iron + folic acid	76 (57.6%)
History of fractures	14 (10.6%): 2 legs; 8 arms, 4 ankles
Gestational diabetes mellitus	4 (3.0%)
Hypothyroid (chronic or gestational)	6 (4.5%)
Chronic diseases	4 (2 Crohn's disease; 1 Gaucher; 1 rheumatoid arthritis)
Smoking history (ever)	9 (6.8%)
Mother with osteoporosis	5 (3.8%)
FN-BMD mean ± SD (range)	0.796 ± 0.11 (0.60–1.08)
FN *T*-scores mean ± SD (range)	[−0.49] ± 0.96 ([−2.3]–2.1)
FN *Z*-scores mean ± SD (range)	[−0.39] ± 0.95 ([−2.1]–2.2)
LS-BMD mean ± SD (range)	0.910 ± 0.34 ([−0.95]–1.44)
LS *T*-scores mean ± SD (range)	[−0.74] ± 1.04 ([−3.2]–3.6)
LS *Z*-scores mean ± SD (range)	[−0.92] ± 1.04 ([−3.2]–3.6)

SD: standard deviation; BMI: body mass index; FN: femoral neck; BMD: bone mineral density; LS: lumbar spine.

**Table 2:** Comparison of means ± SD of demographic characteristics in women ≤30 years (*n* = 73) and women >30 years of age (*n* = 59); significance based on Levene's test for Equality of Variances.

	≤30 years	>30 years	*P* value
Height mean ± SD	162.63 ± 6.38	162.32 ± 6.35	NS
Prepregnancy weight mean ± SD	62.55 ± 13.08	65.78 ± 12.73	NS
Prepregnancy BMI mean ± SD	23.59 ± 4.42	24.89 ± 4.20	NS
Current weight mean ± SD	70.12 ± 13.87	74.02 ± 14.07	NS
Current BMI mean ± SD	26.47 ± 4.71	28.00 ± 4.39	NS
Weight gain mean ± SD	7.58 ± 5.41	9.36 ± 9.28	NS
Gravidity mean ± SD	2.51 ± 1.57	5.95 ± 2.69	0.000
Parity mean ± SD	2.38 ± 1.41	5.83 ± 2.51	0.000
Months lactation mean ± SD	12.04 ± 16.21	45.59 ± 36.08	0.000
Months pregnancy + lactation mean ± SD	32.16 ± 27.06	94.72 ± 54.29	0.000
FN-BMD mean ± SD	0.805 ± 0.11	0.782 ± 0.09	0.031
FN *T*-scores mean ± SD	[−0.401] ± 1.04	[−0.597] ± 0.85	0.023
FN *Z*-scores mean ± SD	[−0.373] ± 1.03	[−0.410] ± 0.84	0.029
LS-BMD mean ± SD	0.921 ± 0.25	0.897 ± 0.43	NS
LS *T*-scores mean ± SD	[−0.907] ± 1.00	[−0.532] ± 1.06	NS
LS *Z*-scores mean ± SD	[−0.830] ± 0.98	[−0.358] ± 1.07	NS

SD: standard deviation; NS: not significant; BMI: body mass index; FN: femoral neck; BMD: bone mineral density; LS: lumbar spine.

**Table 3:** Comparison of bone density variables among primiparous women to the multiparous (≥2 births) women; significance based on Levene's test for Equality of Variances.

	Primiparous (*n* = 27)	Multiparous (*n* = 105)	*P* value
Maternal age mean ± SD (years)	24.9 ± 3.5	31.1 ± 5.8	0.000
Height mean ± SD	162.5 ± 5.5	162.5 ± 6.6	NS
Prepregnancy weight mean ± SD	62.5 ± 15.2	64.4 ± 12.4	NS
Prepregnancy BMI mean ± SD	23.6 ± 5.1	24.3 ± 4.2	NS
Current weight mean ± SD	70.1 ± 14.9	72.3 ± 13.8	NS
Current BMI mean ± SD	26.5 ± 5.2	27.3 ± 4.5	NS
Weight gain mean ± SD	7.6 ± 5.2	8.6 ± 7.9	NS
Gravidity mean ± SD	1.07 ± 0.39	4.81 ± 2.5	0.000
Parity mean ± SD	1.0 ± 0	4.68 ± 2.4	0.000
Months lactation mean ± SD	0	34.1 ± 31.9	0.000
Months pregnancy + lactation mean ± SD	8.5 ± 0	73.4 ± 50.1	0.000
FN-BMD mean ± SD	0.806 ± 0.09	0.791 ± 0.11	NS
FN *T*-scores mean ± SD	[−0.396] ± 0.79	[−0.530] ± 1.0	NS
FN *Z*-scores mean ± SD	[−0.381] ± 0.79	[−0.408] ± 0.99	NS
LS-BMD mean ± SD	0.953 ± 0.11	0.898 ± 0.38	NS
LS *T*-scores mean ± SD	[−0.841] ± 1.0	[−0.715] ± 1.1	NS
LS *Z*-scores mean ± SD	[−0.722] ± 0.93	[−0.594] ± 1.1	NS

^*^SD: standard deviation; NS: not significant; BMI: body mass index; FN: femoral neck; BMD: bone mineral density; LS: lumbar spine.

**Table 4:** Comparison variables among groups subdivided by parity (Group #1: primiparous; Group #2: 2–5 births; Group #3: ≥6 births); significance based on Levene's test for Equality of Variances.

	Group #1 (*n* = 27)	Group #2 (*n* = 69)	Group #3 (*n* = 36)	*P* value
Maternal age mean ± SD (range) in years	24.7 ± 3.6 (20–32)	28.8 ± 5.1 (20–42)	35.8 ± 3.8 (28–46)	0.003
Height mean ± SD	162.2 ± 5.5	162.3 ± 6.9	162.5 ± 6.3	NS
Prepregnancy weight mean ± SD	61.6 ± 15.3	62.3 ± 11.7	68.9 ± 13.0	0.025
Prepregnancy BMI mean ± SD	23.3 ± 5.2	23.6 ± 4.0	25.9 ± 4.0	0.021
Current weight mean ± SD	69.6 ± 15.0	70.0 ± 13.4	77.1 ± 13.5	0.013
Current BMI mean ± SD	26.4 ± 5.3	26.5 ± 4.5	28.9 ± 4.0	0.010
Weight gain mean ± SD	8.0 ± 5.1	8.6 ± 9.2	8.3 ± 4.8	NS
Gravidity mean ± SD	1.0 ± 0	3.3 ± 1.1	7.4 ± 1.8	0.000
Parity mean ± SD	1.0 ± 0.2	3.3 ± 1.2	7.4 ± 1.8	0.000
Months lactation mean ± SD	0	18.4 ± 17.4	64.0 ± 32.1	0.000
Months pregnancy + lactation mean ± SD	8.5 ± 0	45.5 ± 24.3	126.8 ± 42.9	0.000
FN-BMD mean ± SD	0.806 ± 0.09	0.796 ± 0.12	0.784 ± 0.09	NS
FN *T*-scores mean ± SD	[−0.396] ± 0.79	[−0.476] ± 1.1	[−0.583] ± 0.80	NS
FN *Z*-scores mean ± SD	[−0.381] ± 0.79	[−0.399] ± 1.1	[−0.378] ± 0.81	NS
LS-BMD mean ± SD	0.953 ± 0.11	0.940 ± 0.26	0.821 ± 0.54	NS
LS *T*-scores mean ± SD	[−0.841] ± 1.0	[−0.719] ± 1.1	[−0.697] ± 0.95	NS
LS *Z*-scores mean ± SD	[−0.722] ± 0.93	[−0.646] ± 1.1	[−0.489] ± 0.96	NS

SD: standard deviation; NS: not significant; BMI: body mass index; FN: femoral neck; BMD: bone mineral density; LS: lumbar spine.

**Table 5:** Comparison of bone density variables among women with a history of lactation to women who have never breast fed where months of breast-feeding were assessed as a continuous variable.

	Never breast fed (*n* = 35)	Lactation history (*n* = 97)	*P* value
Maternal age mean ± SD (years)	25.3 ± 4.1	31.5 ± 5.7	0.000
Height mean ± SD	163.8 ± 6.0	162.0 ± 6.4	NS
Prepregnancy weight mean ± SD	63.9 ± 16.0	64.0 ± 11.8	NS
Prepregnancy BMI mean ± SD	23.7 ± 5.0	24.3 ± 4.1	NS
Current weight mean ± SD	72.1 ± 16.6	71.8 ± 13.1	NS
Current BMI mean ± SD	26.8 ± 5.2	27.3 ± 4.4	NS
Weight gain mean ± SD	8.2 ± 5.4	8.4 ± 8.0	NS
Gravidity mean ± SD	1.49 ± 1.1	4.97 ± 2.6	0.000
Parity mean ± SD	1.37 ± 0.84	4.85 ± 2.4	0.000
Months lactation mean ± SD	0	36.9 ± 31.6	0.000
Months pregnancy + lactation mean ± SD	11.4 ± 7.1	77.7 ± 49.6	0.000
FN-BMD mean ± SD	0.807 ± 0.10	0.791 ± 0.11	0.014
FN *T*-scores mean ± SD	[−0.383] ± 0.87	[−0.528] ± 0.99	0.013
FN *Z*-scores mean ± SD	[−0.357] ± 0.87	[−0.401] ± 0.98	0.045
LS-BMD mean ± SD	0.963 ± 0.11	0.891 ± 0.39	NS
LS *T*-scores mean ± SD	[−0.757] ± 1.06	[−0.731] ± 1.04	NS
LS *Z*-scores mean ± SD	[−0.649] ± 1.01	[−0.608] ± 1.06	NS

^*^SD: standard deviation; NS: not significant; BMI: body mass index; FN: femoral neck; BMD: bone mineral density; LS: lumbar spine.

**Table 6:** Results in 12 outliers based on *T*-scores and/or *Z*-scores <−2.0 at the LS.

Mean age (years)	26.6 (range: 22–39 with only two >30)
Mean height (cms)	157.4 (range: 145–165)
Mean prepregnancy weight (kgs)	55.4 (range: 41–90)
Mean prepregnancy BMI	22.4 (range: 17–39)
Mean current weight	60.8 (range: 44–85)
Mean current BMI	24.6 (range: 18–37)
Mean weight gain	5.3 (range: [−5]–12)
Mean gravidity	3.4 (range: 1–8)
Mean parity	3.3 (range: 1–8)
Mean number months lactation	24.9 (range: 0–98; three: primiparous; plus one multiparous never breast fed)
Mean number months pregnancy + lactation	52.5 (range: 8.5–166; three: primiparous)
Mean FN-BMD	0.700 (range: 0.605–0.736)
Mean FN *T*-scores	[−1.5] (range: [−1.0]–[−2.2])
Mean FN *Z*-scores	[−1.4] (range: [−1.0]–[−2.1])
Mean LS-BMD	0.800 (range: 0.692–0.831)
Mean LS *T*-scores	[−2.3] (range: [−2.0]–[−3.2])
Mean LS *Z*-scores	[−2.2] (range: [−1.8]–[−3.2])

BMI: body mass index; FN: femoral neck; BMD: bone mineral density; LS: lumbar spine.
